# Nicotinic Acetylcholine Receptor Signaling in Tumor Growth and Metastasis

**DOI:** 10.1155/2011/456743

**Published:** 2011-03-30

**Authors:** Sandeep Singh, Smitha Pillai, Srikumar Chellappan

**Affiliations:** Department of Tumor Biology, H. Lee Moffitt Cancer Center and Research Institute, 12902 Magnolia Drive, Tampa, FL 33612, USA

## Abstract

Cigarette smoking is highly correlated with the onset of a variety of human cancers, and continued smoking is known to abrogate the beneficial effects of cancer therapy. While tobacco smoke contains hundreds of molecules that are known carcinogens, nicotine, the main addictive component of tobacco smoke, is not carcinogenic. At the same time, nicotine has been shown to promote cell proliferation, angiogenesis, and epithelial-mesenchymal transition, leading to enhanced tumor growth and metastasis. These effects of nicotine are mediated through the nicotinic acetylcholine receptors that are expressed on a variety of neuronal and nonneuronal cells. Specific signal transduction cascades that emanate from different nAChR subunits or subunit combinations facilitate the proliferative and prosurvival functions of nicotine. Nicotinic acetylcholine receptors appear to stimulate many downstream signaling cascades induced by growth factors and mitogens. It has been suggested that antagonists of nAChR signaling might have antitumor effects and might open new avenues for combating tobacco-related cancer. This paper examines the historical data connecting nicotine tumor progression and the recent efforts to target the nicotinic acetylcholine receptors to combat cancer.

## 1. Introduction

Smoking is a major risk factor associated with the development and progression of a variety of cancers [1]. Smoking is estimated to account for approximately 4-5 million deaths worldwide and approximately 443,000 deaths each year in the United States alone [2, 3]. Sufficient evidence has accumulated to conclude that tobacco smoking caused cancers not only of the lung, but also of the lower urinary tract including the renal pelvis and bladder, upper aero-digestive tract including oral cavity, pharynx, larynx, and esophagus, and pancreas [2, 4]. Recent lines of evidence have showed that smoking tobacco can also cause cancers of the nasal cavity, paranasal sinus, nasopharynx, stomach, liver, kidney, cervix, uterus, breast, adenocarcinoma of the esophagus, and myeloid leukemia [2]. Of the thousands of chemicals in tobacco smoke, polycyclic aromatic hydrocarbons and nicotine-derived nitrosamines have been identified as the major and potent carcinogens [5, 6]. The metabolites of these agents form DNA adducts and cause mutations in vital genes like Rb, p53, and K-Ras in smokers [7–9]. 

While the induction of these cancers is mediated by tobacco-specific nitrosamines as well as other carcinogens present in the tobacco smoke, it is becoming clear that signaling through the nicotinic acetylcholine receptors contribute to the growth, progression, and metastasis of a variety of cancers. Nicotine, which is the major addictive component of tobacco smoke, acts through nicotinic acetylcholine receptors (nAChR) [9–11], but is not thought to be carcinogenic. The expression of nAChRs in central and peripheral nervous system is associated with smoking dependence and addiction [12]. It was generally believed that nAChRs are only expressed in nervous system and at neuromuscular junctions (muscle type nAChRs). However, the discovery of widespread expression of nAChRs in mammalian cells, including cancers, suggested its direct role in cancer progression [13–15]. This paper deals with certain aspects of nicotinic receptor signaling in nonneuronal cells that lead to increased cell proliferation and survival, angiogenesis, tumor growth, and metastasis.

## 2. Nicotinic Acetylcholine Receptor Expression in Nonneuronal Cells

nAChRs are a complex of five subunits forming hetero- or homopentamers to form a central ion channel [16, 17]. The neuronal nAChRs can be homomeric composed of *α*7, *α*8, or *α*9 subunits or with the combinations of *α*2–*α*6 or *α*10 subunits with *β*2–*β*4 subunits (heteromeric nAChRs). The muscle type nAChRs may be comprised of combinations of *α*1 subunits with *β*1, *γ*, *δ*, or *ε* subunits [18]. Both neuronal as well as muscle nAChR families are found to be expressed in cancer cells [19]. Nicotine mimics acetylcholine by binding as an agonist to *α* subunit of nAChRs [10]. Nicotine binds with higher affinity to heteromeric *α*4*β*2-nAChRs than to *α*7-nAChRs [20]. Higher binding to *α*4*β*2-nAChRs results in desensitization of the receptor, which could be the reason that *α*7-nAChR is the major stimulator of cancer development and progression *in vivo*. In addition to nicotine, tobacco-specific nitrosamines such as 4-(methylnitrosamino)-1-(3-pyridyl)-1-butanone (NNK) can also bind to *α*7-nAchR, and *N*-nitrosonornicotine (NNN) binds to heteromeric *αβ*-nAChRs [21]. The affinity of NNK for the *α*7-nAChR was found to be 1,300 times higher than nicotine, whereas the affinity of NNN for heteromeric *αβ*-nAChRs was 5,000 times higher than that of nicotine [21, 22]. 

Since the discovery of ubiquitous presence of nAChRs in mammalian cells, studies from many laboratories have linked nAChRs with various pathological conditions including tumor growth and angiogenesis [13, 23]. In earlier studies, nicotine was found to stimulate endothelial-cell proliferation via nAChR at concentrations lower than those obtained in blood after smoking [24]. As described in the later part of this paper, many studies have correlated the exposure of nicotine or other tobacco smoke components with induction of pathological neovascularization through the activation of nAChR [23, 25]. Studies from our laboratory have suggested that nicotine can enhance the growth and metastasis of pre-established lung tumors [26]. Altogether, these studies proposed the involvement of tobacco smoke components in various aspects of tumorigenesis and vascular dysfunctions in smokers. Extensive research by many groups has successfully associated the physiological effect of nicotine and its derivatives with the direct activation of nAChRs. Small cell lung carcinoma (SCLC) pulmonary neuroendocrine cells (PNECs) and SCLC cells express high levels of the *α*7-nAChR, whereas heteromeric nAChRs were undetectable [27, 28]. At the same time, both hetero- and homomeric nAChRs are found to be expressed in nonsmall cell lung carcinoma cells of different histologic subtypes [19, 29]. Recently, differential expression pattern of *ACHR* subunit gene was studied in NSCLC patients who were smokers or never smokers. Higher expression of *CHRNA6* and *CHRNB3* combination was correlated with NSCLCs in nonsmokers, whereas lower expression was correlated with NSCLCs in smokers. Additionally, increased expression of *CHRNA1, CHRNA5, and CHRNA7* subunit genes was correlated with short-term exposure to nicotine [30]. Nicotine stimulation contributed towards the growth of human mesothelioma cells. Human biopsies of mesothelioma as well as of normal pleural mesothelial cells were found to express functional *α*7-nAChR [31, 32]. Studies from the Russo laboratory have shown that inhibition of nAChRs by *α*-cobratoxin (*α*-CBT) can inhibit the growth of A549 tumors in immunocompromised mice [33]. These findings strengthen the hypothesis that modulation of nAChRs upon chronic exposure to tobacco may contribute to the development and progression of cancer. In the following sections, we will summarize the findings to support the hypothesis. 

## 3. nAChRs Signaling in Tumor Growth and Survival

Attempts have been made to elucidate the molecular events that mediate nicotine-induced cell proliferation. Activation of nAChR through nicotine or NNK has been found to activate protein kinase C (PKC), the serine/threonine kinase Raf-1, the mitogen-activated kinases ERK1 and ERK2, and the transcription factors FOS, JUN, and MYC through the selective activation of *α*7-nAChR in SCLC [34]. Studies also demonstrated the stereospecificity of nAChRs towards (−)-nicotine. It has been reported that (−)-nicotine stimulated tumor cell proliferation via secretion of the neurotransmitter serotonin, and the growth stimulatory effect of nicotine or NNK could be blocked by selective serotonergic receptor antagonists [27, 35, 36]. In a recent report, the effects of acute and repetitive exposure to nicotine was shown to induce a neuronal-like appearance in N417 SCLC cell line, which produced bigger and more vascularized tumors in mice through activation of CXCR4/CXCL12 axis. A prominent increase in the expression of CXCR4 was observed in nAChR-dependent manner in nicotine-treated cells [37]. NSCLC cell lines from large-cell carcinoma, squamous-cell carcinoma, and adenocarcinoma, all showed the activation of PI3K-AKT pathway and NF-*κ*B activation in response to nicotine or NNK treatment [38, 39]. In addition, frequent loss of the tumor suppressor gene FOXO3a was reported in carcinogen-induced lung adenocarcinoma. In NNK-treated lung cancer cells, restoration of FOXO3a in FOXO3a-deficient cells increases sensitivity to apoptosis caused by a DNA-damaging intermediate of NNK. This study proposed that FOXO3a might play a role in lung adenocarcinoma suppression by providing a protective response to carcinogenic stress [40]. 

**Figure 1:**
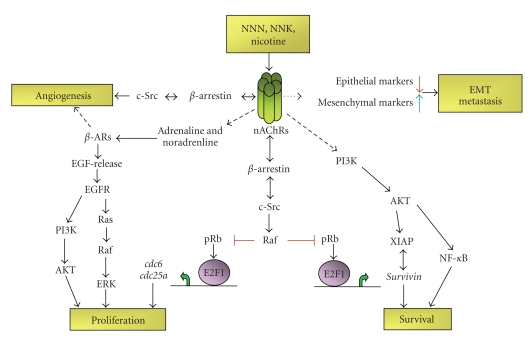
A schematic of nAChR-mediated regulation of diverse tumorigenic processes. nAChRs are activated by tobacco smoke components like NNN, NNK, and nicotine with different affinity. Induced nAChRs activate several downstream signaling pathways involved in cell proliferation, inhibition of apoptosis, metastasis, and angiogenesis in a variety of cancer and primary cells. Agonist binding to nAChR forms complex with *β*-arrestin and Src and results in Raf-1 activation. Activated Raf-1 phosphorylates and inactivates Rb tumor-suppressor-function. These in turn results in E2F-1-mediated transcriptional upregulation of target genes involved in cell proliferation, angiogenesis, and inhibition of apoptosis. Downstream effect of nAChR activation is also indirectly supported by the activation of *β*-adrenergic receptor (*β*-AR) signaling. Nicotine exposure directly results in metastatic dissemination of primary tumor by inducing epithelial to mesenchymal transition (EMT) in cancer cells.

Experiments from our laboratory have shown that nicotine stimulation affects various components of cell cycle regulatory machinery [26, 29, 41]. Exposure to nicotine resulted in activation of Raf-1, induction of cyclin D and cyclin E-associated kinase activity as well as Rb phosphorylation, which led to the dissociation of E2F1 from Rb. Further, it was observed that stimulation with nicotine caused the dissociation of Rb from E2F-responsive proliferative promoters (*cdc6 and cdc25A*), while there were increased amounts of E2F1 bound to them. These molecular events were correlated with increased proliferative effects of nicotine in NSCLC cell lines A549 (human bronchioalveolar carcinoma), NCI-H23, NCI-H441 (lung adenocarcinoma), and NCI-H226 (pleural effusion squamous cell carcinoma) as well as on primary normal human bronchial epithelial cells (NHBEs), small airway epithelial cells (SAECs), human aortic endothelial cells (HAECs), and human microvascular endothelial cells from lung (HMEC-Ls). The mitogenic effects of nicotine were abrogated by *α*7 subunit antagonists, *α*-bungarotoxin, and methylallyl aconitine (MAA), whereas it was unaffected by *α*-lobeline (*α*4*β*2 subunit inhibitor) or dihydro *β*-erythoidine (DH*β*E; *α*3*β*2 and *α*4*β*2 subunit inhibitor), suggesting that *α*7 subunits primarily mediated the mitogenic effects of nicotine in NSCLC cells. We have further illustrated that upon nicotine stimulation, the scaffolding protein *β*-arrestin-1 forms a complex with nonreceptor tyrosine kinase-Src and gets recruited to the nAChRs. Depletion of *β*-arrestin-1 or Src prevented nicotine-induced cell proliferation. These results suggested that *α*7-nAChR-mediated stimulation of cell proliferation is through a *β*-Arrestin-1-Src signaling axis in NSCLC [41]; (see also Figure 1). 

Other than lung cancer, activation of *α*7-nAChR and heteromeric nAChRs expressing *α*3 and *α*5 subunits have been reported in oral and esophageal keratinocytes [22]. Similar to lung cancer cells, NNK was found to bind with high affinity to *α*7-nAChR, whereas NNN was found to bind to heteromeric nAChRs with higher affinity [22]. Esophageal cancer-Het-1A cells stimulated with NNK or NNN showed increased mRNA transcripts and expression of PCNA and Bcl-2, and transcription factors GATA3, NF-*κ*B, and STAT1. However, induction of Ras-Raf-ERK1-ERK2 cascade, the JAK2-STAT3 pathway and NF-*κ*B activation was associated with enhanced cell proliferation through these nitrosamines in immortalized oral epithelial cells [22]. In addition, chronic exposure of nicotine or environmental tobacco smoke on oral keratinocytes selectively upregulated *α*5- and *α*7-nAChR subunits, resulting in intensified signaling responses to nicotine [42]. 

The secreted mammalian Ly-6/urokinase plasminogen activator receptor-related protein-1 (SLURP-1) is recently identified as an endogenous ligand for the *α*7 subunit of the nicotinic acetylcholine receptor (nAChR). The expression levels of SLURP1 and SLURP2 (secreted mammalian Ly-6/urokinase plasminogen activator receptor-related protein-2) were reduced in NNK-treated cells. Transfection of the cells with SLURP1 or SLURP2 cDNA reduced the nitrosamine-induced colony formation in soft agar while inhibiting the growth of NNK-transformed keratinocytes in mouse xenografts. SLURP1 bound to *α*7-nAChR and SLURP2 bound to nAChRs expressing the *α*3 subunit [22, 43]. Similar results were demonstrated recently where HT-29 human colon cancer cells treated with nicotine resulted in increased cell proliferation and a marked reduction in the protein expression of SLURP1 via *α*7-nAChRs activation [44]. Recently, nicotine mediated upregulation of FOXM1 expression was found in primary oral keratinocytes which was associated with induction of genomic instability. A centrosomal protein CEP55 as well as a DNA helicase and putative stem cell marker HELLS, were found to be novel targets of nicotine-induced FOXM1 expression and correlated with oral cancer progression [45].

A role of nAChR has been demonstrated in breast cancer progression as well. Experiments with human mammary epithelial-like MCF10A or cancerous MCF7 cells revealed that treatment of these cells with nicotine enhances the activity of protein kinase C (PKC) alpha with cdc42 as a downstream target for nicotine-induced proliferation and migration [46]. It has also been suggested that nicotine-induced proliferation of human breast cancer cell is dependent on *α*9-nAChR and cyclin D3 expression [47]. The effects of nicotine on a population of cancer stem cells in MCF-7 human breast cancer cells were examined, using aldehyde dehydrogenase (ALDH) as a stem cell marker. This study found that nicotine increases the stem cell population via *α*7-nAChR and the PKC-Notch dependent pathway [48]. 

Apart from direct responses through nAChRs, indirect signaling events may also contribute to nicotine-induced tumor growth and survival. Since nAChRs are cation channels, it can stimulate signaling cascades by the influx of Ca2+ through the opened *α*7-nAChR [49]. Ca2+ channel blockers are shown to significantly reduce DNA synthesis in response to nicotine or NNK in SCLCs [49]. Also, nAChR-mediated systemic increase in stress neurotransmitters, adrenaline, and noradrenaline, which are *β*-adrenergic agonists, are also shown to stimulate *β*-adrenergic receptor-initiated cAMP signaling and transactivation of EGFR cascade through EGF secretion in NNK-treated small airway epithelial cells [50, 51]. Nicotine is found to induce systemic or cellular increase in noradrenaline and significantly enhance the growth and angiogenesis of pancreatic, gastric, and colon cancer-xenografts with increased expression of ERK1-ERK2, COX2, prostaglandin E2, VEGF, and transactivation of *β*-adrenergic as well as EGFR signaling in colon cancer cells [52–55]. Activation of ERK1-ERK2 and STAT3 in response to nicotine has also been reported in bladder cancer cells downstream of nAChRs and *β*-adrenergic receptors [56]. Importantly, apart from nAChRs, direct interaction of NNK with *β*-adrenergic receptor has been proposed as a novel mechanism, which may significantly enhance the high cancer-causing potential of these nitrosamines [50, 57]. Similar to the activation via neurotransmitters, NNK binding to *β*-adrenergic receptor was also found to activate adenylyl cyclase-cAMP-PKA-CREB cascade and transactivation of EFGR [58]. Additionally, an additive effect of estrogen receptors and nAChRs was also demonstrated in promoting the growth of A549 tumors in athymic nude mice. Cotreatment of nicotine and estradiol resulted in increased cell proliferation as well as VEGF secretion from cancer cells, leading to increased tumor growth as well as microvascular density within the tumor [59]. Recently, the chronic exposure to estrogen and NNK was shown to have synergistic effects on cell proliferation and production of noradrenaline and adrenaline, by upregulating *α*7-nAChRs in immortalized small airway epithelial cells [60].

## 4. nAChRs Signaling in Cell Survival and Resistance to Apoptosis

In addition to the effect on tumor growth, epidemiological and clinical data implicate that in patients with cancer, continued smoking causes resistance to therapy by blocking the induction of apoptosis. Various studies have linked the activation of nAChR resulting in inhibition of apoptotic pathways. In SCLC cells, NNK was shown to phosphorylate Bcl-2 at Ser70 which promoted its interaction with c-Myc that significantly enhanced the half-life of the c-Myc protein [61]. This functional cooperation of Bcl2 and c-Myc resulted in promoting cell survival and proliferation. This effect could be blocked by the PKC inhibitor staurosporin, the ERK1-ERK2 inhibitor PD98059 or silencing of MYC [61, 62]. Additionally, mesothelioma cells also showed nicotine-stimulated proliferation through *α*7-nAChR-mediated Ca^2+^-dependent activation of the ERK1-ERK2 cascade and inhibited apoptosis by induction of NF-*κ*B and phosphorylation of BAD at Ser112 (Bcl-2 antagonist of cell death) [32]. In NSCLCs, constitutive activation of AKT is associated with lung cancer cell survival and resistance to chemotherapy and radiation [63]. Similarly, nicotine or NNK exposure displayed AKT-mediated growth and NF-*κ*B-mediated resistance to apoptosis in human airway epithelial cells as well as lung cancer cells [38, 39]. Further, activated AKT could directly phosphorylate Bax *in vitro *in nicotine treated cells. Treatment of cells with the phosphatidylinositol 3-kinase (PI3K) inhibitor LY294002 or specific depletion of AKT was shown to block both nicotine-induced Bax phosphorylation and cisplatin resistance in NSCLC cells [64]. 

In addition to these signaling events, results from our laboratory revealed a significant role for the IAP proteins XIAP and survivin in nicotine-mediated chemoresistance in NSCLCs *in vitro*. Chromatin immunoprecipitation assays demonstrated that nicotine stimulation caused an increased recruitment of E2F1 and concomitant dissociation of retinoblastoma tumor suppressor protein (Rb) from survivin promoter in NSCLC cells [65]. Moreover, ablation of E2F1 levels caused abrogation of survivin expression and protective effects of nicotine against cisplatin-induced apoptosis in A549 cells. In the above study, chemoprotective effect of nicotine was found to be mediated through *α*3*/β*4-nAChR activation and could be abrogated by agonists of these subunits. It was also found that nicotine stimulation enhanced the levels of XIAP at the protein level. Nicotine induces the activation of Akt, which is known to phosphorylate XIAP and prevent its proteasome-mediated degradation [66]. In agreement with this, an Akt inhibitor could abrogate the antiapoptotic effects of nicotine in A549 cells [65]. 

In other studies, the cooperative effect of nicotine and NNK was investigated for their transforming ability in various lung epithelial or cancer cells. Exposure to nicotine or the combination of nicotine and NNK for one week augmented Bcl-2 expression, accompanied by an increased resistance to cisplatin-induced apoptosis [67]. This study also showed that the combination treatment promoted cell proliferation and anchorage-independent growth as compared to NNK exposure alone [67]. In another study, nicotine was demonstrated to mediate prosurvival activity by Mcl-1 phosphorylation. Nicotine-induced Mcl-1 phosphorylation significantly enhanced the half-life of Mcl-1, which conferred long-term survival potential [68]. Specific depletion of Mcl-1 by RNA interference blocked nicotine-stimulated survival and enhanced apoptotic cell death [67]. Nicotine-mediated activation of *α*7-nAChR has also been linked with the expression of PPAR*β*/*δ* protein by inhibiting AP-2*α* protein expression and DNA binding activity to the PPAR*β*/*δ* gene promoter [69]. Sp1 was found to modulate this process. *α*7-nAChR antagonist and short interfering RNA against *α*7-nAChR as well as inhibitors of phosphatidylinositol 3-kinase (PI3K; wortmannin and LY294002) and mammalian target of rapamycin (mTOR; rapamycin) blocked the expression of PPAR*β*/*δ* protein demonstrating a novel mechanism by which nicotine could promote human lung carcinoma cell growth [69]. These studies show that signaling through the nAChRs could promote cell proliferation and survival, utilizing multiple signaling cascades. 

## 5. nAChRs and Tumor Angiogenesis

Angiogenesis, the formation of new blood vessels from pre-existing vasculature, is a complex multistep process involved in a number of physiological processes such as wound healing, embryogenesis and reproduction. In addition, angiogenesis is necessary for the sustained growth of the primary tumor as well as metastatic dissemination. Nicotine has been shown to enhance angiogenesis in many experimental systems and animal models. The proangiogenic activity of nicotine is mediated by nicotinic acetylcholine receptors, particularly *α*7 subunit. The pioneering study by Villablanca (1998) demonstrated the ability of nicotine to induce endothelial cell proliferation [24]. This observation was followed by the elegant studies from the John Cooke's laboratory suggesting a cholinergic pathway for nicotine-induced angiogenesis where they demonstrated complete inhibition of endothelial network formation using nonselective nAChR antagonist mecamylamine in an *in vitro* angiogenesis model [25]. Although several nAChR isoforms are expressed on endothelial cells, a similar inhibition was obtained only with the selective *α*7-nAChR antagonist *α*-bungarotoxin, confirming the specific involvement of *α*7-nAChR. Further, *in vivo* pharmacological inhibition of nAChR and a genetic disruption of *α*7-nAChR expression significantly inhibited inflammatory angiogenesis and reduced ischemia-induced angiogenesis and tumor growth. They also provided anatomic and functional evidence for nicotine-induced angiogenesis and arteriogenesis when they observed that nicotine accelerated the growth of tumor and atheroma in association with increased neovascularization [23]. 

Nicotine increased endothelial-cell growth and tube formation *in vitro,* and accelerated fibrovascular growth *in vivo*. In a mouse model of hind-limb ischemia, nicotine increased capillary and collateral growth, and enhanced tissue perfusion. These effects of nicotine were mediated through nicotinic acetylcholine receptors at nicotine concentrations that are pathophysiologically relevant and suggested a possible role for the endothelial production of nitric oxide, prostacyclin, and vascular endothelial growth factor [70–74]. Nicotine has been demonstrated to stimulate postnatal angiogenesis, having an antiapoptotic effect on endothelial cells. It was observed that nicotine stimulated postnatal vasculogenesis on endothelial progenitor cells (EPCs) [75]. The effect of nicotine on EPC survival was significantly enhanced under serum starvation. Furthermore, the antiapoptotic effect of nicotine was blocked completely by nicotinic acetylcholine receptor (nAChR) antagonist hexamethonium bromide [75]. 

Recent studies have shown that apart from cigarette smoking, exposure to secondhand smoke also could induce angiogenesis. A positive correlation between secondhand smoke exposure and concentrations of nicotine in the body was established after analyzing twenty-two studies measuring the biological effects of nicotine [76]. Further, it was found that the levels of nicotine exposure from secondhand smoke were comparable to those of active smokers. In a mouse model where Lewis lung cancer cells were implanted subcutaneously into mice, which were then exposed to sidestream smoke (SHS) or clean room air and administered vehicle or mecamylamine (an inhibitor of nAChR); SHS significantly increased tumor size, weight, capillary density, VEGF, and MCP-1 levels, and circulating endothelial progenitor cells (EPC). Mecamylamine partially inhibited the effects of SHS on these angiogenic processes and nearly abolished the effects of SHS on tumor capillary density suggesting that nicotine mediated the effects of SHS on tumor angiogenesis and growth [77].

Several recent studies have implicated that nicotine-induced angiogenesis could be mediated by growth stabilization and transmigration of endothelial progenitor cells (EPC) [75, 78, 79]. Nicotine accelerated the growth of syngenic colon cancer CMT93 cells when grown subcutaneously in mice by inducing angiogenesis via bone marrow derived EPCs [78]. To determine if the angiogenic effects of nicotine is mediated by EPC mobilization, Heeschen et al. used a model of mouse parabiosis and found that nicotine enhances EPC mobilization into the vasculature of the ischemic tissue. This effect may be due to the direct actions of nicotine on EPC proliferation, migration and/or mobilization as suggested by *in vitro* models [80] and plasma markers used in the investigation [79]. They also noticed that in the absence of acute ischemia, nicotine did not stimulate EPC mobilization. The activation of nAChRs in response to ischemia induced the release of proangiogenic factors like VEGF and stem cell derived factor-1, both of which are regulated by hypoxia, which in turn facilitates EPC mobilization [81]. Evidence from another study also demonstrated that nicotine promotes angiogenesis via stimulation of nAChR-dependent endothelial cell migration. nAChR antagonism not only abolished nicotine-induced human microvascular endothelial cells (HMVEC) migration but also abolished migration induced by bFGF and attenuated migration induced by VEGF. Transcriptional profiling identified gene expression programs which were concordantly regulated by all 3 angiogens (nicotine, VEGF, and bFGF), a notable feature of which includes corepression of thioredoxin-interacting protein (TXNIP), endogenous inhibitor of the redox regulator thioredoxin. Furthermore, TXNIP repression by all 3 angiogens induced thioredoxin activity. Interestingly, nAChR antagonism abrogates growth factor (VEGF- and bFGF-) mediated induction of thioredoxin activity suggesting the requirement of nAChR activation in endothelial cell migration, a key angiogenesis event [82]. 

The proangiogenic effects of nicotine have been found to be mediated by *α*7-nAChR on endothelial cells by activating ERK/MAP kinase pathway, PI3 kinase/Akt pathway, and NF-*κ*B [23, 25, 83, 84]. Further, nicotine has been shown to induce the proangiogenic factors like VEGF and HIF-1*α* in NSCLC cell lines [85]. Pharmacologically blocking nAChR-mediated signaling cascades, including the Ca2+/calmodulin, Src, protein kinase C, PI3K/Akt, MAPK/ERK1/2, mTOR pathways, significantly attenuated nicotine-induced upregulation of HIF-1*α*. These proangiogenic and invasive effects of nicotine were partially abrogated by depleting HIF-1*α* using siRNA techniques. Additionally, nicotine could promote angiogenesis of gastric cancers by upregulating COX2 and VEGFR2 [86]. Nicotine also enhanced the activity of matrix metalloproteinase 2 and 9 and expression of plasminogen activators in a COX2 and VEGFR2-dependent manner. The proangiogenic effect of nicotine has been shown to be dependent on Src activity by our laboratory [41]. The inhibition of Src, using chemical inhibitors or siRNA has been shown to inhibit endothelial cell proliferation, migration, and angiogenic tubule formation on matrigel. As mentioned earlier, studies from our laboratory suggest that the scaffolding protein *β*-arrestin-1 causes the activation of Src. Oligomeric complex comprising of nAChR, *β*-arrestin-1, and Src is vital for nAChR signaling. In addition, depletion of *β*-arrestin-1 caused abrogation of endothelial cell proliferation and angiogenic tubule formation [29, 41]. These data suggest that nicotine behaves in a manner analogous to growth factors and induces cell cycle progression in endothelial cells. 

## 6. nAChRs in EMT and Tumor Metastasis

Epithelial to mesenchymal transition (EMT) is a biological process that allows a polarized epithelial cell, which normally interacts with the basement membrane through its basal surface, to undergo multiple biochemical changes with a signature of more advanced and less differentiated cancer that allow it to assume a mesenchymal phenotype. This enhanced migratory capacity, invasiveness, resistance to apoptosis, and greatly increased production of ECM components [87–89]. This process results in degradation of basement membrane and the formation of a mesenchymal like cell, which can migrate away from the epithelial layer in which it originated [88]. Epithelial to mesenchymal transition (EMT) is involved in tumor progression from noninvasive tumor cells into metastatic carcinomas. Recent studies from our laboratory demonstrated that nicotine can induce invasion and migration in cell lines derived from lung cancer, breast cancer, and pancreatic cancer via *α*7-nAChR-mediated signal transduction pathways [90]. The proinvasive effects of nicotine were mediated by *α*7-nAChR in lung cancer cells while *α*7-nAChR and Dh*β*E sensitive nAChRs mediated invasion of breast cancer cells. Nicotine was also found to inhibit anoikis in lung airway epithelial cells. Further, nicotine could induce changes in gene expression consistent with EMT. Long-term treatment of lung cancer and breast cancer cells with nicotine was found to diminish levels of epithelial markers namely *β*-catenin and E-cadherin and upregulate mesenchymal proteins like fibronectin and vimentin, indicative of disruption of cell-cell contacts and increased motility [90]. 

In addition to facilitating EMT, nicotine and NNK have been shown to affect various aspects of tumor cell invasion and migration. For example, both nicotine and NNK are shown to promote the invasion of NSCLC by phosphorylation of *μ* and m-calpains [62]. Several lines of evidence show that calpain-mediated proteolysis mediates various aspects of cell physiology including cell migration and invasion. Nicotine was found to induce phosphorylation of both *μ* and m-calpains via *α*7-nAChR; the binding of nicotine to *α*7-nAChR in turn was found to activate Src and PKC-iota, leading to enhanced invasion and migration of NSCLC cell line H1299. Similarly, NNK also could promote invasion and migration through phosphorylation of *μ* and m-calpains in a *α*7-nAChR-dependent fashion [62]. 

Several observations in patients suggest that those exposed to tobacco carcinogens are more likely to develop larger, more vascularized tumors with a high propensity for metastatic spread and resistance to chemotherapy [90]. About 30% of lung cancer patients who are smokers continue to smoke after they have been diagnosed [91], which might result in increased adverse medical consequences such as increased tumor progression, development of a second cancer, greater recurrence, greater cancer-related mortality and reduced quality of life [92, 93]. While these studies demonstrate a role for tobacco carcinogens in the initiation, growth, and progression of cancers, the relative contribution of nicotine by itself to these processes is not well explored. A recent study from our laboratory demonstrated that nicotine by itself can induce the growth and metastasis of tumors in immunocompetent mice, independent of other tobacco carcinogens [26]. Nicotine administered either intraperitonially or by commercially available transdermal patches could substantially promote tumor growth. Similar effects were observed on implanted tumors as well as tumors induced by tobacco carcinogen, NNK. Furthermore, mice exposed to nicotine showed significantly enhanced lung metastasis as well as tumor recurrence after surgical removal of the primary tumor, indicating that nicotine can enhance the growth and metastasis of pre-established lung tumors [26]. As mentioned earlier, repetitive exposure to nicotine on SCLC-N417 cells resulted in neuronal-like appearance along with increased adhesion to the extracellular matrix. These changes were accompanied by enhanced migration through collagen matrices and adhesion to and transmigration across lymphatic endothelial cell monolayers [37]. 

Accumulating evidence from epidemiological studies suggest a strong association between smoking and pulmonary metastatic disease in women with breast cancer [94]. In a murine model of metastatic mammary cell cancer, cigarette smoke exposure was associated with an increase in the total pulmonary metastatic burden providing experimental support for an adverse effect of smoking on the metastatic process and suggesting a possible mechanism for smokers' increased breast cancer mortality [95]. In addition, it was observed that cigarette smoking was correlated with increased lymph node metastases at mastectomy in women older than 50 years of age suggesting that tobacco usage may potentiate the early spread of malignant disease [96]. Although numerous studies have indicated the role of nicotine exposure in tumor promotion, little is known about the molecular mechanisms by which nicotine promoted breast tumor development, especially on the metastatic process of breast cancer. At least four different subunits of nAChRs including *α*5, *α*7, *α*9, and *β*4 are shown to be expressed in breast cancer cells [46]. It has been demonstrated that in addition to proliferative effect, nicotine promoted migration of breast cell lines (mammary epithelial cell line MCF10A and breast cancer cell line MCF7) through a signaling cascade involving PKC activation and its downstream effector cdc42 [46]. Exposure to nicotine has shown to increase the expression of *α*9-nAChR in breast cancer cells [47, 97]. Studies using a soft agar transforming assay and a mouse xenograft model demonstrated that noncancerous human breast epithelial cell line, MCF10A, could be neoplastically transformed by exposure to either a cigarette smoke condensate or the tobacco specific carcinogen, NNK [98, 99]. In a recent study, *α*9-nAChR expression was silenced in MDA-MB-231 breast cancer cells which resulted in reduced proliferation and tumorigenic potential in both *in vitro* and *in vivo* assays, indicating the role of *α*9-nAChR in breast carcinogenesis [100]. 

Cigarette smoking has recently been recognized as a risk factor for gastric cancer [101] and long-term exposure of nicotine-induced EMT like changes in gastric cancer cell lines by activating Erk/5-Lox signaling pathway [102]. A study on the association between cigarette smoking and pancreatic cancer showed that smokers had a significantly higher risk (70%) of developing pancreatic cancer compared to nonsmokers [103–105]. Accumulating evidence suggests that nicotine induces expression of osteopontin, a secreted phosphoprotein that confers on cancer cells a migratory phenotype and activates signaling pathways that induce cell survival, proliferation, invasion, and metastasis. Rats exposed to cigarette smoke showed a dose-dependent increase in pancreatic osteopontin expression. In addition, analysis of cancer tissues from invasive pancreatic ductal adenocarcinoma (PDA) patients, the majority of whom were smokers, showed the presence of significant amounts of osteopontin in malignant ducts and the surrounding pancreatic acini [106]. Further studies suggested that nicotine contributes to PDA metastasis by inducing MMP9 and VEGF expression and osteopontin mediated these effects [107]. An osteopontin isoform, OPNc, is selectively inducible by nicotine and is highly expressed in PDA tissues from smokers which induced the expression of monocyle chemoattractant protein (MCP-1) indicating a proinflammatory role of nicotine [108]. Altogether, these results suggest that nicotine plays a key role in the regulation of the complex cellular cascades that modulate cell adhesion, invasion, and migration leading to metastasis. 

## 7. Discussion and Conclusions

Tobacco smoking is a well-documented risk factor for many cancers. As summarized in Figure 1, nicotine, the principal addictive component of tobacco smoke, as well as other nitrosamines have been found to act through nAChRs on nonneuronal cells to facilitate tumor growth, angiogenesis, metastasis, survival, and chemoresistance by regulating diverse signaling pathways. Binding of agonist to nAChR facilitates the complex formation between the receptor, scaffolding protein *β*-arrestin and tyrosine kinase Src. Activation of Src was found to be important for cancer as well as endothelial cell proliferation and angiogenic tube formation *in vitro*. Proliferative effect of nAChR-activation was also supported by indirect stimulation of *β*-adrenergic receptor (*β*-AR) signaling. Further, chemotherapy-induced apoptosis was found to be blocked by nicotine-induced survivin expression as well as NF-*κ*B activation. Activation of nAChR is also correlated with EMT-like changes and metastatic dissemination of primary tumor cells. Given the ability of nicotine to affect various aspects of tumor growth and metastasis, antagonists of nAChR signaling might be beneficial in controlling the growth and progression of tumors. Recently, alpha cobratoxin (*α*-CbT) has been shown to block the growth of a variety of NSCLC and mesothelioma cell lines both *in vitro* and *in vivo *[109, 110]. The most striking effect of *α*-CbT was its ability to effectively inhibit the metastatic potential of lung cancer cells transplanted into nude mice, indicating the possibility of using nAChR antagonists as adjuvant therapy in preventing metastatic spread. At the same time, the potential side effects of nAChR antagonists on the brain and central nervous system need to be investigated before using them as a viable drug for combating lung cancer. Moreover, the direct role of nicotine alone on several aspects of tumorigenesis raises the need to revisit the potential tumor promoting effects of nicotine-replacement therapy. Also, the modulation effects of secondhand smoke on nAChRs require detailed investigation in the future.
